# Pretreatment plasma vitamin D and response to neoadjuvant chemotherapy in breast cancer: evidence from pooled analysis of cohort studies

**DOI:** 10.1097/JS9.0000000000002142

**Published:** 2024-11-18

**Authors:** Chi Shu, Qian Yang, Jun Huang, Xuan Xie, Hong Li, Hong Wu, Xin Wang, Xin Chen, Yuping Xie, Yanhong Zhou, Yazhou He, Chuan Xu

**Affiliations:** a Department of General Surgery, Division of Vascular Surgery, West China Hospital, Sichuan University, Chengdu, China; b Department of Oncology and Cancer Institute, Sichuan Academy of Medical Sciences, Sichuan Provincial People’s Hospital, University of Electronic Science and Technology of China, Chengdu, China; c Department of Oncology and Department of Epidemiology and Medical Statistics, West China School of Public Health and West China Fourth Hospital, Sichuan University, Chengdu, China; d Department of General Surgery, West China Hospital, Sichuan University, Chengdu, China; e Colorectal Cancer Center, West China Hospital, Sichuan University, Chengdu, China; f Department of Laboratory Medicine, West China Hospital, Sichuan University, Chengdu, China

**Keywords:** breast cancer, meta-analysis, neoadjuvant chemotherapy, pathological response, vitamin D

## Abstract

**Background:**

Biological evidence has revealed antitumor effect of vitamin D, but whether it could predict the response to neoadjuvant chemotherapy (NAC) in breast cancer (BC) patients remains inconclusive. The aim was to investigate the association between pretreatment vitamin D level and response to NAC and subsequent survival outcomes in BC patients.

**Materials and methods:**

The authors systematically searched the Medline, Embase, Cochrane Library, and Web of Science databases and clinical trial registries to identify relevant articles from inception to 8 October 2024. Eligible studies investigating the associations between pretreatment plasma vitamin D and response to NAC in BC patients were selected according to the predefined criteria, with the study characteristics extracted by two reviewers. The primary outcome was pathological complete response (pCR), while overall pathological response and event-free survival (EFS) were adopted as secondary outcomes. Summary effect estimates of odds ratios (ORs) or hazard ratios (HRs) with 95% CIs were pooled using a random-effects model. Subgroup and sensitivity analyses were performed based on study characteristics and methodological quality.

**Results:**

Six retrospective cohort studies involving 1291 BC patients were included. The authors observed a significant association between pretreatment vitamin D deficiency and 50% increased odds of non-pCR after NAC (OR=1.50, 95% CI: 1.11–2.03, *P*=0.008) with no heterogeneity (*I* ^2^=0%). The authors also identified a significant association of vitamin D with the overall pathological response (OR=1.33, 95% CI: 1.01–1.75, *P*=0.046). A similar association with EFS (HR=1.27, 95% CI: 0.92–1.75, *P*=0.139) was also noted although the effect estimate was not statistically significant. Sensitivity analyses based on methodological quality showed consistent findings.

**Conclusion:**

Pretreatment vitamin D deficiency is associated with an inferior response to NAC in BC patients. Our meta-analysis advocates further prospective studies with large sample sizes before vitamin D supplementation could be administered to improve NAC response and subsequent prognosis of BC patients.

## Introduction

Highlights

- Pretreatment vitamin D deficiency was associated with 50% increased odds of non-pCR to neoadjuvant chemotherapy in breast cancer patients.
- Combining estimates from studies using varied criteria for pathological response, lower vitamin D level was also associated with inferior overall response, and potential worse event-free survival.
- Our findings laid the foundation for future prospective investigations and randomized controlled trials exploring the effects of vitamin D supplementation on improving neoadjuvant chemotherapy response.

Breast cancer (BC) is the most prevalent female malignancy and the fifth leading cause of cancer-related deaths worldwide^1^. In recent years, neoadjuvant chemotherapy (NAC) has become a standard care for locally advanced BC, in pursuit of an increased breast conservation rate by reducing tumor size^2–4^. Response to NAC has been robustly linked to higher R0 resection rate and favorable subsequent survival outcomes^5,6^. However, tumor heterogeneity and variations in patient characteristics impose major challenges in predicting patients’ response to NAC^7–9^. Identifying early predictors of response to NAC has important clinical implications in informing treatment efficacy and, therefore, improving prognosis.

Vitamin D is an essential nutrient that plays key roles in a wide spectrum of biological processes. In particular, the antitumor effect of vitamin D has been increasingly recognized, including regulating proliferation, differentiation, apoptosis, angiogenesis, invasion, and metastasis of tumor cells^10–12^. Preclinical studies have indicated that vitamin D could enhance the efficacy of BC chemotherapy by synergizing or amplifying the cytotoxic activity of antineoplastic agents, such as anthracyclines and taxanes^13–15^. Specifically, it acts on the transporter to regulate the distribution of the drug in the body, thereby increasing the concentration of the drug in cancer cells^16^. Adding vitamin D to paclitaxel could reduce the expression of chemo-resistance marker genes such as multidrug resistance complex 1^17^. Evidence also reported that vitamin D deficiency was associated with a higher risk of side effects such as chemotherapy-induced peripheral neuropathy^18^. These findings shed light upon the possible role of vitamin D in augmenting the NAC effectiveness within a limited timeframe of a neoadjuvant setting.

Whilst aforementioned biological evidence supported a possible beneficial effect of vitamin D on response to NAC for BC patients, findings of population-level investigations in the association between pretreatment circulating vitamin D and pathological response to NAC remained inconclusive, which might be attributed to the relatively small sample sizes for single clinical investigations regarding NAC for BC patients. In addition, there has been a dearth of a comprehensive review on these evidence despite the potential clinical relevance. We therefore set out to systematically review published evidence and to estimate the association between pretreatment plasma vitamin D level and response to NAC in BC patients with a markedly improved statistical power by performing a meta-analysis.

## Materials and methods

### Study design and search strategies

We searched the Medline, Embase, Cochrane Library, and Web of Science databases and clinical trial registries from inception to 8 October 2024, using combined keywords including ‘breast cancer’ AND (‘vitamin D’ OR ‘25-hydroxyvitamin D’) AND ‘neoadjuvant’. We described the search strategies in detail in the Supplemental Table 1 (Supplemental Digital Content 1, http://links.lww.com/JS9/D556). The references of relevant articles were manually screened to identify additional eligible studies. Also, the investigators of relevant conference abstracts were traced separately to identify any subsequent potential publications. This work has been reported in line with Preferred Reporting Items for Systematic Reviews and Meta-Analyses (PRISMA)^19^ checklist presented in Supplemental Digital Content 2, http://links.lww.com/JS9/D557 and flow diagram in Supplemental Digital Content 3, http://links.lww.com/JS9/D558 and Assessing the methodological quality of systematic reviews (AMSTAR)-2 guidelines^20^ (Supplemental Digital Content 4, http://links.lww.com/JS9/D559). The protocol was registered on the International Prospective Register of Systematic Reviews (PROSPERO, CRD42024532891).

### Inclusion and exclusion criteria

Eligible studies were expected to meet the following inclusion criteria: 1) studies focusing on females with histologically confirmed BC; 2) investigations with sample sizes no less than 30; 3) studies that assayed pretreatment circulating vitamin D level at BC diagnosis as the primary exposure; 4) studies that adopted measures for NAC response, such as pCR, or long-term (≥12 months) survival outcomes for particular events, for example, tumor recurrence or death from BC after NAC as the outcomes; 5) investigations with study designs including clinical trials, prospective or retrospective cohort studies, and case–control studies. Studies were excluded if they were published as conference abstracts, comments, case reports, letters, or editorials. During the screening process, two reviewers (C.S. and Q.Y.) independently screened titles and abstracts, with disagreements resolved by consensus or consultation with a third reviewer (Y.H.). We documented the source of included patients in each study, namely the patient database, registry, or study cohort, along with the time-span of patient enrollment to ascertain whether there were overlapping patients included by other eligible studies. Where the name of the data source was not provided, we noted the hospital or institute of patient enrollment instead. If multiple studies included patients from the same data source or institution within an overlapped time span, only the latest publication was included.

### Data extraction and quality assessment

Data items regarding basic characteristics were extracted from each eligible study by two independent reviewers (C.S. and Q.Y.), including the first author, publication year, data source, country, study design, molecular subtypes, clinical tumor stage, vitamin D assessment measure, pretreatment vitamin D level, vitamin D deficiency cut-off value, outcome measures, and sample sizes. The details of data extraction items are presented in the Supplemental Table 2 (Supplemental Digital Content 1, http://links.lww.com/JS9/D556). We also documented effect estimates reported by each study including odds ratios (ORs) or hazard ratios (HRs) with corresponding 95% CI (or event numbers where effect estimates could be derived). HRs with 95% CIs were not obtained from the survival curves.

We evaluated the methodological quality of the included studies using the Newcastle–Ottawa Scale (NOS)^21,22^. The scoring criteria are presented in the Supplemental Method 1 (Supplemental Digital Content 1, http://links.lww.com/JS9/D556). Studies with the NOS scores no less than 7 were deemed high quality. Any discrepancies were solved by discussion with a senior clinician and epidemiologist (Y.H. and C.X.).

### Outcome definition

We employed the pCR as the primary outcome, which was defined as the absence of invasive tumor cellularity in the resected breast tumor specimen and axillary lymph nodes after NAC (residual in situ cancer without invasive cancer was considered to have a pCR^23^). Previous evidence has established robust associations between achieving pCR and improved long-term survival outcomes in BC^5,6^; and subsequently pCR has been officially listed as a recommended surrogate outcome by the Food and Drug Administration (FDA) and European Medicines Agency (EMA)^24,25^. We also defined an overall pathological response to NAC by combining treatment responses using a combined criteria, that is patients showing responses determined by achieving pCR or response in any of other criteria including residual cancer burden (RCB)^26^ score (RCB ≤1 was defined as responders) and Miller-Payne grading (MPG, grade ≥4 was defined as responders)^27^. The detailed definitions for each outcome are summarized in the Supplemental Method 2 (Supplemental Digital Content 1, http://links.lww.com/JS9/D556). As for survival outcomes, an event-free survival (EFS), where events included local recurrence, distant metastasis, or death from cancer, was adopted to evaluate any associations with long-term prognosis.

### Statistical analysis

All statistical analysis was conducted using the ‘meta’ package of R statistical software (version 4.1.3; https://www.R-project.org/). We conducted evidence synthesis to estimate the pooled effect estimates of vitamin D deficiency (defined as pretreatment serum vitamin D<20 ng/ml based on the recommendation from Institute of Medicine [IOM]^28^) on each outcome. The between-study heterogeneity in the reported effect estimates was estimated using the *I* ^2^ statistic^29^. Given the inherent heterogeneity cross included studies, we performed meta-analysis using a random-effects model with the DerSimonian and Laird method to calculate pooled odds or hazard ratios and their 95% CIs. Forest plots were used to visualize each individual and the pooled effect estimates with their 95% CIs. An OR or HR >1 indicated a detrimental effect of vitamin D deficiency on the odds of response to NAC.

Subgroup analyses were preplanned based on prior evidence that suggested possible varied vitamin D effects across different patient strata, including biological relevance (tumor stage^30^, molecular subtype^31,32^), treatment regimen^33^, patient ethnicity^34^, and cut-off thresholds of vitamin D level^35^. We assessed publication bias by visual evaluation of the funnel plot symmetry and by conducting the Egger’s^36^ tests where a *P*-value<0.10 indicated the presence of a significant small study effect. Sensitivity analyses were conducted by excluding studies of lower quality (NOS<7) from the meta-analysis. All statistical tests were 2-sided, with a *P*-value <0.05 deemed statistically significant.

## Results

### Study selection and quality assessment

**Figure 1:**
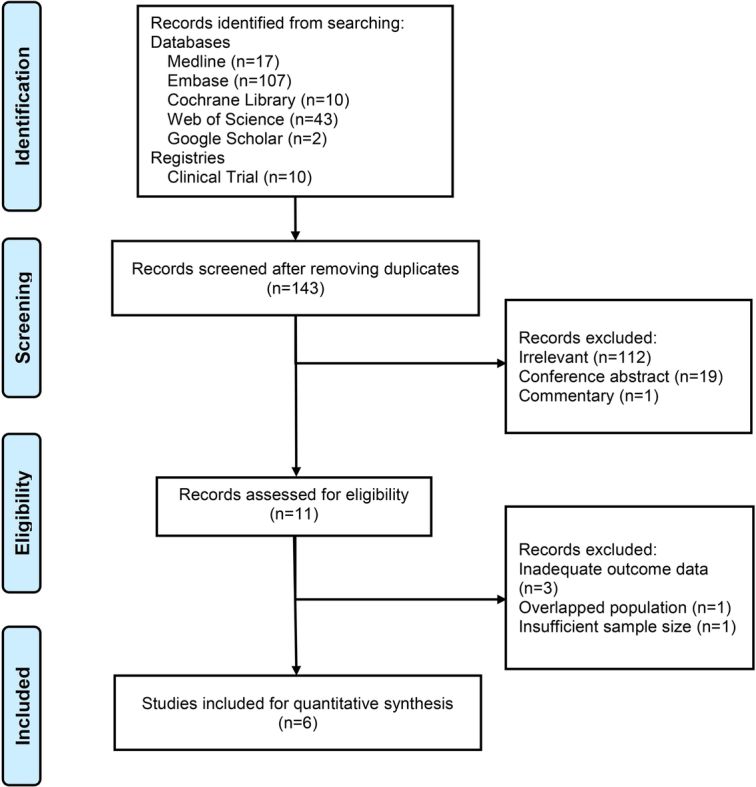
Flowchart of the studies selection process.

Our initial literature search yielded 189 citations. After excluding 46 duplicates, 132 studies were removed by reviewing the titles and abstracts based on our inclusion criteria. After that, a total of eleven studies were reviewed in full text^5,37–46^. Of which, one study was excluded due to a duplicated study population^5^, one study was excluded for a small sample size (<30)^43^, three studies were excluded for lacking available outcome data^44–46^ (details of the flow diagram presented in Fig. 1).

**Figure 2:**
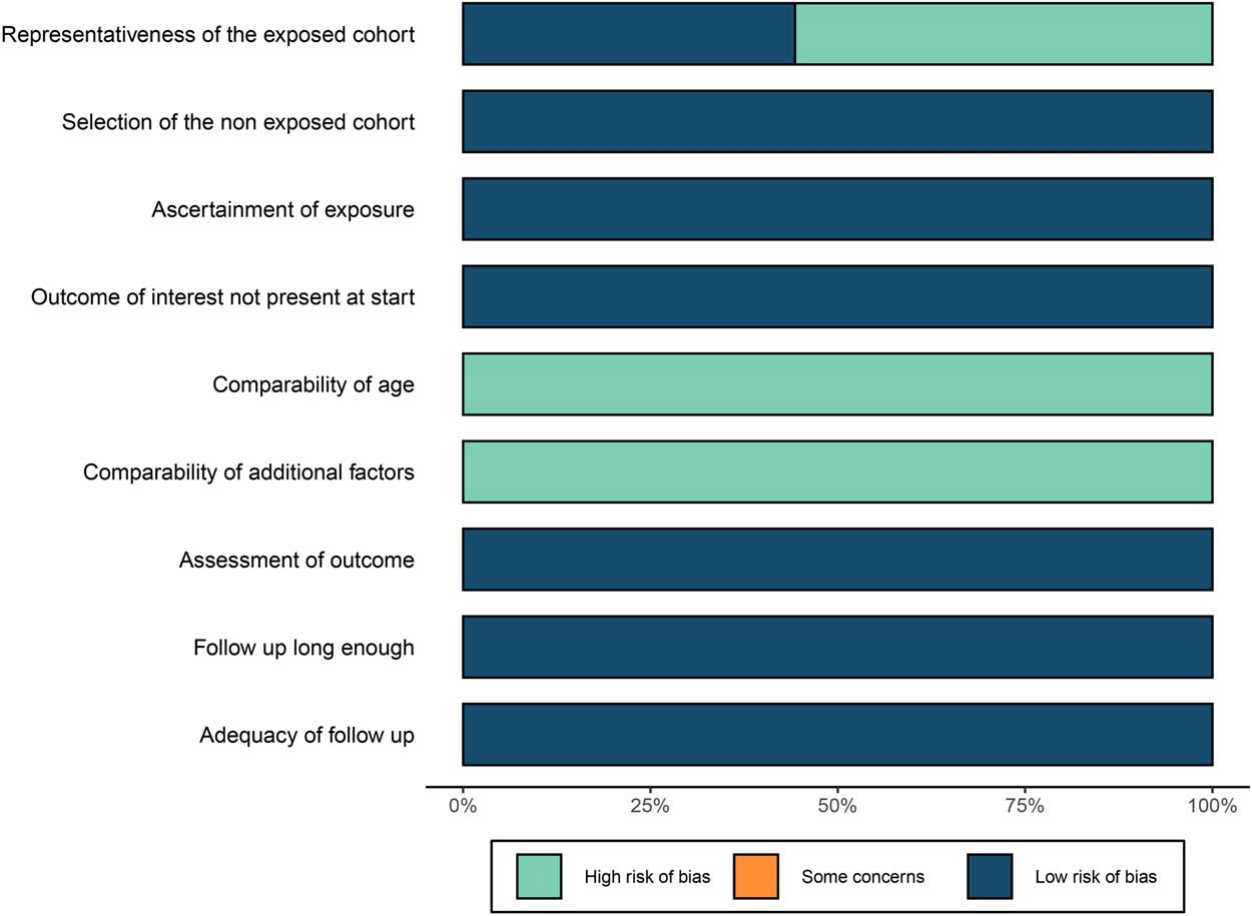
Risk of bias plot on pathological complete response (pCR).

Six studies with a total of 1291 BC patients were included^37–42^. All studies were retrospective in design. Agents used in the NAC regimens adopted by these studies included anthracycline-based drugs, taxane, and trastuzumab. All included studies examined serum vitamin D levels prior to NAC. The basic characteristics of eligible studies are presented in Table 1 and the Supplemental Table 3 (Supplemental Digital Content 1, http://links.lww.com/JS9/D556), where all six included studies enrolled patients from independent data sources with no duplicate. Three studies were assigned with high methodological quality (NOS≥7)^37,38,40^. The detailed results of the quality assessment are presented in the Supplemental Tables 4–6 (Supplemental Digital Content 1, http://links.lww.com/JS9/D556). The risk of biases for the included studies was likely to occur in aspects of patient selection and comparability across groups (Fig. 2 and Supplemental Fig. 1A,B of Supplemental Digital Content 1, http://links.lww.com/JS9/D556). The quality of evidence for included studies (AMSTAR-2) is presented in Supplemental Digital Content 4, http://links.lww.com/JS9/D559.

**Table 1:** Main characteristic of included studies.

Author	Year	Country	Data source	Study design	Molecular subtypes	Tumor stage	Vitamin D assessment	Vitamin D deficiency cut-off value, ng/ml	Outcomes	OR or HR (95% CI)	Sample sizes
Clark^37^	2014	America	I-SPY1 Trial	R	HER-2 negative	II–III	RIA	<20 vs. ≥20 or per 1-unit decrease^a^	RCB EFS	OR^a^: 1.01 (0.96–1.05) OR: 0.75 (0.14–2.19) HR: 1.30 (0.57–2.94)	82
Charehbili^38^	2016	Netherlands	NEOZOTAC Trial	R	HER-2 negative	II–III	NR	Per 1-unit decrease^a^	pCR	OR^a^: 1.00 (0.99–1.01)	169
Kim^39^	2018	Korea	Severance Hospital	R	NS	I–III	RIA	<20 vs. ≥20	pCR EFS	OR: 1.54 (0.92–2.60) HR: 1.00 (0.46–2.17)	374
Viala^40^	2018	America, France	Cancer Center in Montpellier and Lowa	R	NS	I–III	ECLIA, MFA	<20 vs. ≥20	pCR EFS	OR: 1.64 (1.02–2.66) HR: 1.11 (0.67–1.67)	327
Atci^41^	2021	Turkey	Istanbul City Hospital	R	NS	III	ECLIA	<20 vs. ≥20	MPG	OR: 0.63 (0.27–1.47)	89
Tokunaga^42^	2022	Japan	Kyushu Cancer Center	R	NS	I–III	ELISA	<20 vs. ≥20	pCR EFS	OR: 1.27 (0.71–2.28) HR: 2.28 (1.12–5.03)	250

ECLIA, electrochemiluminescence immunoassay; EFS, event-free survival, including recurrence-free survival (RFS), disease-free survival (DFS), progression-free survival (PFS) and time to distant recurrence (TTDR); ELISA, Enzyme linked immunosorbent assay; HER-2, human epidermal growth factor receptor 2; HR, hazard ratio; MFA, multiplex flow immunoassay; MPG, Miller-Payne grading (MPG ≥ 4 was defined as responders); NR, not reported; NS, not specified; OR, odds ratio; pCR, pathological complete response (No invasive tumor cell in the resected breast tumor specimen and axillary lymph nodes after neoadjuvant chemotherapy); R, retrospective; RCB, residual cancer burden (RCB ≤ 1 was defined as responders); RIA, radioimmunoassay.

^a^ Vitamin D as a continuous variable.

### Associations between vitamin D and NAC response

**Figure 3:**
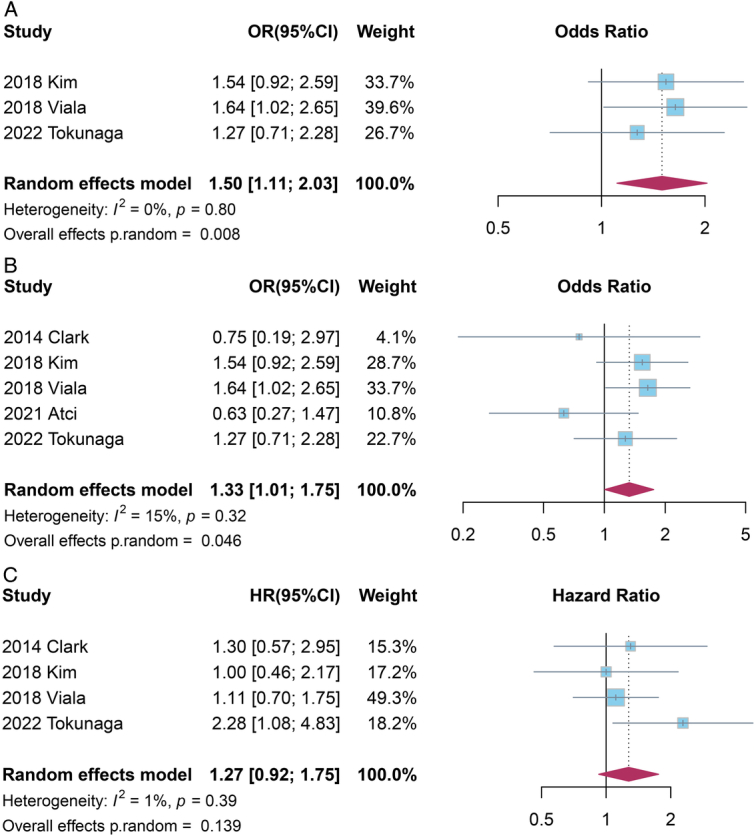
A Forest plot of the effects of vitamin D deficiency on not achieving pathological complete response (pCR); B Forest plot of the effects of vitamin D deficiency on no overall pathological response; C Forest plot of the effects of vitamin D deficiency on event-free survival (EFS).

Among the included studies, three included stage I–III tumors and used pCR as the primary outcome to measure the pathological response to NAC. As shown in Fig. 3A, by conducting meta-analysis, we observed a significant association between pretreatment vitamin D deficiency and an increased odd of non-pCR (OR=1.50, 95% CI: 1.11–2.03, *P*=0.008). Of note, effect estimates reported by these three studies showed good consistency where no between-study heterogeneity was detected (*I* ^2^=0%). As for overall pathological response (Fig. 3B) with combined measures of pCR, RCB and MPG, five studies were included and the meta-analysis identified a significant association of vitamin D deficiency with inferior response to NAC (OR=1.33, 95% CI: 1.01–1.75, *P*=0.046). Of note, we observed a moderate level of heterogeneity (I^2^=15%) for this outcome with a broader definition of response. Visual inspection of the contour-enhanced funnel plot (Supplemental Fig. 2A,B of Supplemental Digital Content 1, http://links.lww.com/JS9/D556) did not identify any evident asymmetry, and the Egger’s test did not find any significant small study effects or publication bias (pCR: *P*=0.104; overall pathological response: *P*=0.819).

Four studies reported EFS in BC patients receiving NAC. As shown in the forest plot (Fig. 3C), with mild heterogeneity revealed (*I* ^2^=1%), BC patients with pretreatment vitamin D deficiency tended to experience inferior EFS, although the association was not statistically significant (HR=1.27, 95% CI: 0.92–1.75, *P*=0.139). The funnel plot for EFS (Supplemental Fig. 2C of Supplemental Digital Content 1, http://links.lww.com/JS9/D556) was also deemed symmetrical, with an Egger’s test presenting no evidence of small study effects (*P*=0.585).

### Subgroup and sensitivity analysis

**Figure 4:**
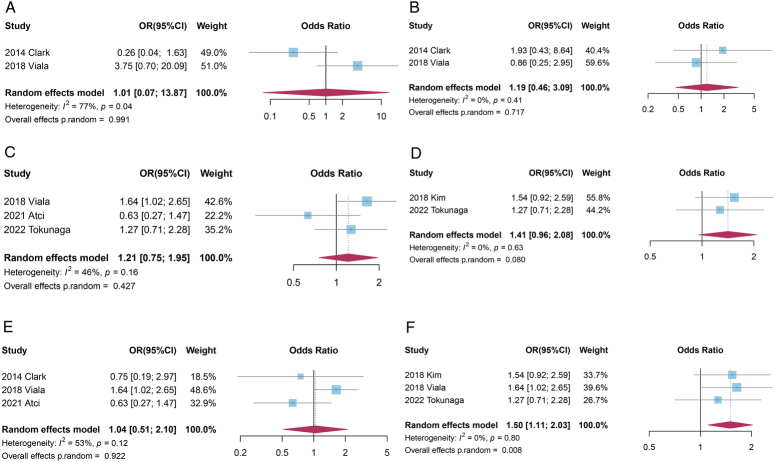
Subgroup analysis on the overall pathological response (vitamin D as a dichotomous variable). A Subgroup plot with HR-positive; B Subgroup plot with HR negative; C Subgroup plot with Chemotherapy including trastuzumab; D Subgroup plot with Asian; E Subgroup plot with European; F subgroup plot with stages I–III; HR-positive: Either estrogen receptor (ER) or progesterone receptor (PR) is positive; HR negative: Both estrogen and progesterone receptors are negative.

We conducted a subgroup meta-analysis using a random-effects model based on different study characteristics, including molecular subtype (hormone receptor, HR), treatment regimen, patient ethnicity, and clinical tumor stage. Outside meta-analysis on studies including stages I–III patients where pCR was adopted as the primary outcome, we did not find any significant associations between vitamin D deficiency and response to NAC, although all the pooled estimates showed concordant directions of effect that indicated potential harmful roles of vitamin D deficiency on NAC response (vitamin D as a dichotomous variable, with forest plots presented in Fig. 4A–F). Similar results were also found in terms of overall pathological response (vitamin D as a continuous variable, Supplemental Fig. 3 of Supplemental Digital Content 1, http://links.lww.com/JS9/D556), as well as EFS (regimen, ethnicity, and tumor stage, Supplemental Fig. 4A–D of Supplemental Digital Content 1, http://links.lww.com/JS9/D556).

We conducted sensitivity analysis on response to NAC (Supplemental Fig. 5A of Supplemental Digital Content 1, http://links.lww.com/JS9/D556) and EFS (Supplemental Fig. 5B of Supplemental Digital Content 1, http://links.lww.com/JS9/D556) outcomes by including only studies with high methodological quality (NOS≥7), and the direction of effects remained consistent (response: OR=1.46, 95% CI: 0.85–2.52, *P*=0.170; EFS: OR=1.27, 95% CI: 0.92–1.75, *P*=0.139). In addition, two studies analyzed the association using vitamin D level of a continuous scale^37,38^, our meta-analysis failed to observe a significant association between lower vitamin D (per 1 unit) and response to NAC (OR=1.00, 95% CI: 0.99–1.01, *P*=0.924, Supplemental Fig. 6 of Supplemental Digital Content 1, http://links.lww.com/JS9/D556).

## Discussion

### Summary of main findings

To our best knowledge, this is the first systematic review and meta-analysis investigating the association between pretreatment vitamin D and response to NAC in BC patients. Our findings revealed a significant association of vitamin D deficiency with inferior response to NAC. Subgroup and sensitivity analysis found generally consistent evidence, though causality cannot be inferred from these observational data.

### Comparison with existing literature and interpretation

Previous epidemiological evidence has robustly linked low circulating vitamin D level to escalated BC risk^47–49^. In addition, vitamin D deficiency was more prevalent among patients with advanced stages and unfavorable clinicopathological characteristics, and could also influence overall survival for BC patients^50,51^. These findings were consistent with biological evidence underpinning the potential antitumor functions of vitamin D. Recent evidence has shed light upon the potential synergetic effect of vitamin D on chemotherapy drugs and its protective effect against chemotherapy side effects. For example, vitamin D could intensify the combined effect of 5-aminolevulinic acid-based photodynamic therapy (ALA-PDT) and paclitaxel on tumor growth and apoptosis in BC cells^14^. Also, studies showed that vitamin D supplementation provided protective effects against doxorubicin (DOX)-induced cardiotoxicity in BC patients^52,53^. Vitamin D insufficiency, on the other hand, was associated with chemotherapy-induced peripheral neuropathy from paclitaxel for early-stage BC^18^. Therefore, current biological evidence linked vitamin D level to response to NAC of BC and laid the foundation for our study to further investigate its predictive role in clinical practice.

Our meta-analysis provided the first population-level evidence supporting the linkage between pretreatment vitamin D deficiency and inferior response to NAC in BC patients. By pooling effect estimates, our study overcame the primary limitation of previous studies—insufficient statistical power from limited sample sizes—in the setting of NAC. We employed pCR as the primary outcome measuring pathological response to NAC given that it has been officially listed as a surrogate outcome by FDA and EMA^24,25^ for accelerated drug approval, and has been widely adopted as the primary outcome by many published trials. Notably, two of the included studies^37,41^ utilized MPG or RCB criteria to evaluate tumor response, where formulas were applied to created scores to measure the residual tumor burden. These two studies included relatively small sample sizes and adding their estimates to our meta-analysis yielded a significant association between vitamin D deficiency and poor overall response, although an expected elevated moderate heterogeneity was observed for this secondary outcome. With a broader definition of treatment response using various criteria such as RCB and MPG, the pooled estimate still indicated a significant association between vitamin D deficiency and inferior response, which further strengthened the robustness of our findings. We also noticed that these two studies reported point estimates of opposite direction, although the effects were nonsignificant. In addition to the different criteria used to define pathological response, they included BC patients with more advanced tumor stages (II–III), leading to possible lower response rates indicated by previous evidence^40,42^, and subsequently contributing to the heterogeneous effect estimates. The small sample sizes (<100) also yielded a greater extend of variability to their estimates, and their point estimates should be interpreted with caution. EFS was also investigated as a secondary outcome in our study. Due to a limited number of outcome events, we failed to identify a significant impact of vitamin D on EFS, although a consistent direction of association was detected. Previous observational evidence had uncovered a favorable impact of vitamin D level on overall and disease-free survival for all BC patients^54^, and this was expected to be further validated among BC patients receiving NAC by conducting more prospective studies with larger sample sizes.

Another point worth mentioning was the dichotomized definition and arbitrary threshold selection of vitamin D deficiency. Although all the included studies chose a widely recommended threshold (<20 ng/ml), vitamin D levels and deficiency thresholds can vary depending on the assay used, geography, and population characteristics^55,56^. Moreover, epidemiological evidence demonstrated that dichotomy could lead to loss of information with subsequent impaired statistical power and possible biases^57^. Our systematic review only found two studies^37,38^ that reported the regression coefficients where vitamin D level was modeled in a continuous scale, and null effects were identified from the underpowered meta-analysis. Future efforts were merited to explore the possible dose-response relationship between vitamin D level and response to NAC.

Of note, our extensive literature search identified no prospective studies yet on this topic, leading to all the included studies in our meta-analysis being retrospective in design, which introduced a risk of bias, particularly in patient selection and comparability, across different studies. Among the eligible studies, the primary source for risk of bias based on the NOS score was the lack of comparability, where crude ORs were used as effect estimates with covariates unadjusted. Consequently, one of the three studies with high risk (NOS<7)^39,41,42^ reported an reversed direction of effect^41^, although the result was not statistically significant. To mitigate the risk of selection bias, the included high-quality (NOS≥7) studies enrolled patients from multiple centers, while adjusting for common covariates in multivariable analyses^37,38,40^. Based on that, we conducted sensitivity analysis by excluding studies with low NOS scores, and observed a consistent direction of effect, which further strengthened the robustness of our findings. However, due to the retrospective nature, our findings could provide initial evidence on the association between vitamin D and NAC response, and support a pressing need for prospective studies in the future to confirm this finding.

Anthracycline-based or taxane-based therapies are commonly prescribed in the current regimen of NAC^58^, which were also adopted by studies included in this meta-analysis. For human epidermal growth factor receptor 2 (HER-2) positive patients, trastuzumab would also be added. We conducted subgroup analysis based on treatment regimen and found the nonsignificant effect of vitamin D among studies including trastuzumab for HER-2 positive patients. However, due to data availability, we were unable to perform separate analysis on HER-2 positive patients, which merited future investigation in whether vitamin D was less predictive of response to targeted therapies as current biological evidence has been primarily focusing on chemotherapeutic agents. Scientific evidence indicated that vitamin D intensified the effects of paclitaxel through down-regulated B-cell lymphoma-2 (Bcl-2) expression and upregulated the expression of Bcl-2 associated X (Bax) and cleaved caspase-3 in BC cells, thereby promoting the apoptosis of BC tumor cells^14,17^. With respect to other molecular subtypes, we conducted stratified analyses by hormone receptor (HR) status, but with only two studies eligible, we did not identify any significant effects of vitamin D among either HR+ or HR− patients. There has been evidence showing that lower vitamin D level was associated with unfavorable BC molecular subtypes, that is triple-negative tumors^35,59^. Vitamin D might also influence the therapeutic effect in BC patients with certain molecular subtypes, such as estrogen receptor (ER) and HR-positive tumors^60–62^. These findings pointed to a potential modifying effect of molecular subtypes on the association between vitamin D and treatment response. Owing to limited data, however, we were unable to assess possible effect modification by conducting subgroup analyses. More studies with granular subgroup data are warranted in the future to further clarify these effects.

### Strengths and limitations of the study

We have performed, to our best knowledge, the first systematic review and meta-analysis on the association between pretreatment vitamin D and response to NAC in BC patients. However, there were several limitations that should be noted for current evidence. Firstly, although our meta-analysis has provided pooled effect estimates with much improved statistical power, relatively small sample sizes, especially for specific subgroups, could limit the credibility of our findings. Given the unavailable individual-level data, we were unable to adjust the possible confounders such as comorbidities and lifestyles (e.g. obesity and physical activity), which were associated with vitamin D level and could influence response to NAC. Moreover, there has been a lack of data for patients of particular molecular subtypes such as triple-negative BC. Secondly, the retrospective nature of included studies incurred a risk of biases, and the observed association could not be interpreted as causality. Our study calls for more prospective studies with larger sample sizes to further validate the association. Thirdly, the included studies used the same threshold to dichotomize vitamin D, although the measured level could vary across diverse populations and different measuring techniques, which remained to be further investigated with more granular data accumulated in the future. Fourthly, although the funnel plots and Egger’s test did not identify significant biases from small study effects, the statistical tests were underpowered due to the small number of included studies (n<10) and the results should be interpreted with caution. Finally, due to limited data, current evidence was unable to address the possible dose-response effect of vitamin D on NAC response. As such, future efforts are imperative to conduct large-scale prospective cohort studies with well-defined subgroups of various molecular subtypes to reassess this association.

### Clinical implications and future directions

Vitamin D deficiency is highly prevalent worldwide and persists as a potential shared risk factor for many conditions including cancer, posing a major threat to human health^63^. BC patients are more susceptible to vitamin D deficiency in comparison to the general population^64,65^. Moreover, a significant decrease of the vitamin D level has been widely observed after the completion of NAC in BC patients^66,67^. Previous evidence has observed an adverse impact of lower plasma vitamin D level on survival outcomes of BC patients, on top of which our study provided further insights that vitamin D could potentially sensitize patients to NAC.

Our meta-analysis of retrospective cohorts calls for more prospective studies to investigate the potential causality between vitamin D and response to NAC. On the basis of a robust association, advanced approaches such as mendelian randomization could be performed to explore the potential causality between vitamin D level and clinical outcomes in BC patients, and randomized controlled trials (RCTs) that investigate the effects of vitamin D supplementation with varied doses and time schedules on improving response to NAC in BC patients are also expected in the future. Also, as circulating vitamin D can be readily modified at a low cost, our findings highlighted the possibility of improving response to NAC for BC patients via vitamin D supplementation and laid the groundwork for future well-designed clinical investigations exploring varied doses of vitamin D supplementation for BC patients receiving NAC. Given the limited sample size of current studies^68^, whether intense pretreatment vitamin D supplementation could compensate for the adverse impact on response to NAC or whether it would be too late still needs to be verified by future population-based prospective studies.

## Conclusion

Our study suggested an association between pretreatment vitamin D deficiency and reduced response to NAC in BC patients, although causality could not be inferred from current evidence with observational design. Therefore, active surveillance on vitamin D level as a biomarker before and during NAC were warranted for BC patients. However, as a safe intervention at a low cost, the causal effect of vitamin D supplementation to improve treatment response could only be proved by future RCTs. Also, our meta-analysis supports a compelling need for future prospective studies with larger sample sizes and well-designed subgroups to strengthen current evidence.

## Ethical approval

Not applicable.

## Consent

Not applicable.

## Source of funding

This work was supported by the National Key Research and Development Program of China (grant no. 2023YFC3402100), the National Natural Science Foundation of China (grant no. 92259102 and 82103918), the Natural Science Foundation of Sichuan Province (grant no. 2024NSFSC0057 and 2025NSFSC0554).

## Author contribution

Q.Y., C.S., J.H., X.X., H.L., X.W., X.C., Y.X., Y.Z., C.X., and Y.H.: acquisition, analysis, or interpretation of data; Q.Y., C.S., Y.H., and C.X.: drafting of manuscript; C.S. and X.X.: statistical analysis; Y.H. and C.X.: study supervision. All co-authors contributed in critical revision of the manuscript for important intellectual content.

## Conflicts of interest disclosure

The authors declare no conflicts of interest.

## Research registration unique identifying number (UIN)

1. Name of the registry: International Prospective Register of Systematic Reviews (PROSPERO).
2. Unique identifying registration number: CRD42024532891.
3. Hyperlink to the registration: https://www.crd.york.ac.uk/prospero/#recordDetails.

## Guarantor

Yazhou He.

## Data availability statement

All data in this article can be shared upon reasonable request to the corresponding author.

## Provenance and peer review

Not commissioned, externally peer-reviewed.

## Supplementary Material

SUPPLEMENTARY MATERIAL
