# Supplementary material for: Pretreatment plasma vitamin D and response to neoadjuvant chemotherapy in breast cancer: evidence from pooled analysis of cohort studies
Source: Int J Surg. 2024 Nov 18;110(12):8126–35. doi: 10.1097/JS9.0000000000002142 (PMC11634150; doi:10.1097/JS9.0000000000002142)
Supplement: SUPPLEMENTARY MATERIAL [file js9-110-8126-s001.docx]

**Pretreatment plasma vitamin D and response** **to** **neoadjuvant chemotherapy in breast cancer: evidence from pooled analysis of cohort studies**

**Supplemental Table 1** Strategy for the literature search

**Supplemental Table 2** Data extraction form of included studies

**Supplemental Table 3** Additional baseline characteristics of the six included studies in the meta-analysis

**Supplemental Table 4** Quality assessment of literature on overall pathological response

**Supplemental Table 5** Quality assessment of literature on pathological complete response (pCR)

**Supplemental Table 6** Quality assessment of literature on event-free survival (EFS)

**Supplemental Method 1** Newcastle-Ottawa Scale (NOS) risk of bias scoring guide

**Supplemental Method** **2** The definition of outcomes

**Supplemental Figure** **1** A Risk of bias plot on overall pathological response; B Risk of bias plot on event-free survival (EFS)

**Supplemental Figure 2** A Funnel plot on pathological complete response (pCR); B Funnel plot on overall pathological response; C Funnel plot on event-free survival (EFS)

**Supplemental Figure** **3** Subgroup analysis on overall pathological response (vitamin D as a continuous variable)

**Supplemental Figure** **4** Subgroup analysis on event-free survival (EFS). A Subgroup plot with Chemotherapy including trastuzumab, B Subgroup plot with Asian, C Subgroup plot with European, D subgroup plot with Stage I-III

**Supplemental Figure** **5** A Sensitivity analysis on overall pathological response; B Sensitivity analysis on event-free survival (EFS).

**Supplemental Figure 6** Forest plot of the summarized results regarding overall pathological response (vitamin D as a continuous variable)

**Supplemental Table 1 Strategy for the literature search**

| **Medline / Embase / Cochrane Library** | |
| --- | --- |
| No. | Search |
| 1. | exp “Breast Neoplasms”/ |
| 2. | (“breast cancer” OR “breast carcinoma” OR “breast carcinomas” OR “breast malignant neoplasm” OR “breast malignant neoplasms” OR “breast malignant tumor” OR “breast malignant tumors” OR “breast neoplasms” OR “breast neoplasms” OR “breast tumor” OR “breast tumors” OR “cancer, breast” OR “cancer, mammary” OR “cancer of breast” OR “cancer of the breast” OR “cancers, mammary” OR “carcinoma, breast” OR “carcinoma, human mammary” OR “carcinomas, breast” OR “carcinomas, human mammary” OR “human mammary carcinoma” OR “human mammary carcinomas” OR “human mammary neoplasm” OR “human mammary neoplasms” OR “malignant neoplasm of breast” OR “malignant tumor of breast” OR “mammary cancer” OR “mammary cancers” OR “mammary carcinoma, human” OR “mammary carcinomas, human” OR “mammary neoplasm, human” OR “mammary neoplasms, human” OR “neoplasm, breast” OR “neoplasm, human mammary” OR “neoplasms, breast” OR “neoplasms, human mammary” OR “tumor, breast” OR “tumors, breast” ).tw. |
| 3. | 1 or 2 |
| 4. | exp “Vitamin D”/ |
| 5. | ("Receptors, Calcitriol" OR "Sunshine Vitamin").tw OR exp “Ergocalciferols”/ OR ("Calciferols" OR "Vitamin D 2" OR "Vitamin D2" OR "D2, Vitamin" OR "Ergocalciferol").tw |
| 6. | exp Cholecalciferol/ |
| 7. | ("Calciol" OR "(3 beta,5Z,7E)-9,10-Secocholesta-5,7,10(19)-trien-3-ol" OR "Vitamin D 3" OR "Vitamin D3" OR "Cholecalciferols").tw |
| 8. | ("25-Hydroxyvitamin D" OR "Calcidiol" OR "25(OH)D" OR "25-OH Vitamin D" OR "25-Hydroxycholecalciferol" OR "25 Hydroxycholecalciferol" OR "25-(OH)-D").tw |
| 9. | exp "25-Hydroxyvitamin D 2"/ |
| 10. | ("25 Hydroxyvitamin D 2" OR "25-Hydroxyergocalciferol" OR "25 Hydroxyergocalciferol" OR "25-Hydroxyvitamin D2" OR "25 Hydroxyvitamin D2" OR "9,10-Secoergosta-5,7,10(19),22-tetraene-3 beta,25-diol" OR "Ercalcidiol" OR "25-Hydroxycalciferol" OR "25 Hydroxycalciferol" OR "25-OH Vitamin D2" OR "25(OH)D2" OR "25-OH-D2").tw |
| 11. | exp Calcifediol/ |
| 12. | ("25-Hydroxyvitamin D 3" OR "25 Hydroxyvitamin D 3" OR "25-Hydroxycholecalciferol Monohydrate" OR "25 Hydroxycholecalciferol Monohydrate" OR "Monohydrate, 25-Hydroxycholecalciferol" OR "25-Hydroxyvitamin D3" OR "25 Hydroxyvitamin D3" OR "Calcifediol, (3 beta,5E,7E)-Isomer" OR "Calcifediol Anhydrous" OR "Anhydrous, Calcifediol" OR "Dedrogyl" OR "Hidroferol" OR "Calcifediol, (3 alpha,5Z,7E)-Isomer" OR "Calderol" OR "25-OH D3" OR "25-(OH)-D3").tw |
| 13. | exp "1,25-dihydroxyergocalciferol"/ |
| 14. | ("1,25-dihydroxyvitamin D2" OR "1,25-(OH)2D2" OR "1,25-dihydroxyergocalciferol, (1alpha,3beta,5Z,7E,22E)-isomer" OR "PRI-1906" OR "1 alpha,25-dihydroxyvitamin D2" OR "1,25-(OH)2-D2" OR "1,25-Dihydroxycholecalciferol" OR "Calcitriol (D2)").tw |
| 15. | exp "1,25-dihydroxylumisterol (3)"/ |
| 16. | ("1 alpha,25-dihydroxylumisterol (3)" OR "1,25-(OH)2-D3" OR "1,25-Dihydroxycholecalciferol" OR "Calcitriol (D3)" OR "1,25-Dihydroxy-3-epi-vitamin D3").tw |
| 17. | 4 or 5 or 6 or 7 or 8 or 9 or 10 or 11 or 12 or 13 or 14 or15 or16 |
| 18. | exp “Neoadjuvant Therapy”/ |
| 19. | (“chemoradiation, neoadjuvant” OR “chemoradiation therapy, neoadjuvant” OR “chemoradiation treatment, neoadjuvant” OR “chemoradiotherapy, neoadjuvant” OR “chemotherapy, neoadjuvant” OR “chemotherapy treatment, neoadjuvant” OR “neoadjuvant chemoradiation” OR “neoadjuvant chemoradiation therapies” OR “neoadjuvant chemoradiation therapy” OR “neoadjuvant chemoradiation treatment” OR “neoadjuvant chemoradiation treatments” OR “neoadjuvant chemoradiations” OR “neoadjuvant chemoradiotherapies” OR “neoadjuvant chemoradiotherapy” OR “neoadjuvant chemotherapies” OR “neoadjuvant chemotherapy” OR “neoadjuvant chemotherapy treatment” OR “neoadjuvant chemotherapy treatments” OR “neoadjuvant radiation” OR “neoadjuvant radiation therapies” OR “neoadjuvant radiation therapy” OR “neoadjuvant radiation treatment” OR “neoadjuvant radiation treatments” OR “neoadjuvant radiations” OR “neoadjuvant radiotherapies” OR “neoadjuvant radiotherapy” OR “neoadjuvant systemic therapies” OR “neoadjuvant systemic therapy” OR “neoadjuvant systemic treatment” OR “neoadjuvant systemic treatments” OR “neoadjuvant therapies” OR “neoadjuvant therapy” OR “neoadjuvant treatment” OR “neoadjuvant treatments” OR “radiation, neoadjuvant” OR “radiation therapy, neoadjuvant” OR “radiation treatment, neoadjuvant” OR “radiotherapy, neoadjuvant” OR “systemic therapy, neoadjuvant” OR “systemic treatment, neoadjuvant” OR “therapy, neoadjuvant” OR “therapy, neoadjuvant chemoradiation” OR “therapy, neoadjuvant radiation” OR “therapy, neoadjuvant systemic” OR “treatment, neoadjuvant” OR “treatment, neoadjuvant chemoradiation” OR “treatment, neoadjuvant chemotherapy” OR “treatment, neoadjuvant radiation” OR “treatment, neoadjuvant systemic” OR “neoadjuvant hormone therapy” OR “neoadjuvant targeted therapy”).tw |
| 20. | (Preoperative OR “Preoperative Therap*” OR “Preoperative Treatment*” OR “Primary Systemic Therap*” OR “Primary Therap*” OR “Initial Treatment*” OR “Initial Therap*” OR “Therapeutic Priming” OR “Downstaging Therap*” OR “Tumor Shrinkage Therap*” OR “Novel Auxiliary treatment*” OR “Novel Auxiliary therap*”).tw |
| 21. | 18 or 19 or 20 |
| 22. | 3 and 17 and 21 |
| **Web of Science** | |
| No. | Search |
| 1 | TS= (“Breast Neoplasms” OR “breast cancer” OR “breast carcinoma” OR “breast carcinomas” OR “breast malignant neoplasm” OR “breast malignant neoplasms”OR “breast malignant tumor” OR “breast malignant tumors” OR “breast neoplas” OR “breast neoplasms” OR “breast tumor” OR “breast tumors” OR “cancer, breast” OR “cancer, mammary” OR “cancer of breast” OR “cancer of the breast” OR “cancers, mammary” OR “carcinoma, breast” OR “carcinoma, human mammary” OR “carcinomas, breast” OR “carcinomas, human mammary” OR “human mammary carcinoma” OR “human mammary carcinomas” OR “human mammary neoplasm” OR “human mammary neoplasms” OR “malignant neoplasm of breast” OR “malignant tumor of breast” OR “mammary cancer” OR “mammary cancers” OR “mammary carcinoma, human” OR “mammary carcinomas, human” OR “mammary neoplasm, human” OR “mammary neoplasms, human” OR “neoplasm, breast” OR “neoplasm, human mammary” OR “neoplasms, breast” OR “neoplasms, human mammary” OR “tumor, breast” OR “tumors, breast”) |
| 2 | TS= (“Vitamin D” OR "Receptors, Calcitriol" OR "Sunshine Vitamin" OR “Ergocalciferols” OR "Calciferols" OR "Vitamin D 2" OR "Vitamin D2" OR "D2, Vitamin" OR "Ergocalciferol" OR Cholecalciferol OR "Calciol" OR "(3 beta,5Z,7E)-9,10-Secocholesta-5,7,10(19)-trien-3-ol" OR "Vitamin D 3" OR "Vitamin D3" OR "Cholecalciferols" OR "25-Hydroxyvitamin D" OR "Calcidiol" OR "25(OH)D" OR "25-OH Vitamin D" OR "25-Hydroxycholecalciferol" OR "25 Hydroxycholecalciferol" OR "25-(OH)-D" OR "25-Hydroxyvitamin D 2" OR "25 Hydroxyvitamin D 2" OR "25-Hydroxyergocalciferol" OR "25 Hydroxyergocalciferol" OR "25-Hydroxyvitamin D2" OR "25 Hydroxyvitamin D2" OR "9,10-Secoergosta-5,7,10(19),22-tetraene-3 beta,25-diol" OR "Ercalcidiol" OR "25-Hydroxycalciferol" OR "25 Hydroxycalciferol" OR "25-OH Vitamin D2" OR "25(OH)D2" OR "25-OH-D2" OR Calcifediol OR "25-Hydroxyvitamin D 3" OR "25 Hydroxyvitamin D 3" OR "25-Hydroxycholecalciferol Monohydrate" OR "25 Hydroxycholecalciferol Monohydrate" OR "Monohydrate, 25-Hydroxycholecalciferol" OR "25-Hydroxyvitamin D3" OR "25 Hydroxyvitamin D3" OR "Calcifediol, (3 beta,5E,7E)-Isomer" OR "Calcifediol Anhydrous" OR "Anhydrous, Calcifediol" OR "Dedrogyl" OR "Hidroferol" OR "Calcifediol, (3 alpha,5Z,7E)-Isomer" OR "Calderol" OR "25-OH D3" OR "25-(OH)-D3" OR "1,25-dihydroxyergocalciferol" OR "1,25-dihydroxyvitamin D2" OR "1,25-(OH)2D2" OR "1,25-dihydroxyergocalciferol, (1alpha,3beta,5Z,7E,22E)-isomer" OR "PRI-1906" OR "1 alpha,25-dihydroxyvitamin D2" OR "1,25-(OH)2-D2" OR "1,25-Dihydroxycholecalciferol" OR "Calcitriol (D2)" OR "1,25-dihydroxylumisterol(3)" OR "1 alpha,25-dihydroxylumisterol(3)" OR "1,25-(OH)2-D3" OR "1,25-Dihydroxycholecalciferol" OR "Calcitriol (D3)" OR "1,25-Dihydroxy-3-epi-vitamin D3") |
| 3 | TS= (“Neoadjuvant Therapy” OR “chemoradiation, neoadjuvant” OR “chemoradiation therapy, neoadjuvant” OR “chemoradiation treatment, neoadjuvant” OR “chemoradiotherapy, neoadjuvant” OR “chemotherapy, neoadjuvant” OR “chemotherapy treatment, neoadjuvant” OR “neoadjuvant chemoradiation” OR “neoadjuvant chemoradiation therapies” OR “neoadjuvant chemoradiation therapy” OR “neoadjuvant chemoradiation treatment” OR “neoadjuvant chemoradiation treatments” OR “neoadjuvant chemoradiations” OR “neoadjuvant chemoradiotherapies” OR “neoadjuvant chemoradiotherapy” OR “neoadjuvant chemotherapies” OR “neoadjuvant chemotherapy” OR “neoadjuvant chemotherapy treatment” OR “neoadjuvant chemotherapy treatments” OR “neoadjuvant radiation” OR “neoadjuvant radiation therapies” OR “neoadjuvant radiation therapy” OR “neoadjuvant radiation treatment” OR “neoadjuvant radiation treatments” OR “neoadjuvant radiations” OR “neoadjuvant radiotherapies” OR “neoadjuvant radiotherapy” OR “neoadjuvant systemic therapies” OR “neoadjuvant systemic therapy” OR “neoadjuvant systemic treatment” OR “neoadjuvant systemic treatments” OR “neoadjuvant therapies” OR “neoadjuvant therapy” OR “neoadjuvant treatment” OR “neoadjuvant treatments” OR “radiation, neoadjuvant” OR “radiation therapy, neoadjuvant” OR “radiation treatment, neoadjuvant” OR “radiotherapy, neoadjuvant” OR “systemic therapy, neoadjuvant” OR “systemic treatment, neoadjuvant” OR “therapy, neoadjuvant” OR “therapy, neoadjuvant chemoradiation” OR “therapy, neoadjuvant radiation” OR “therapy, neoadjuvant systemic” OR “treatment, neoadjuvant” OR “treatment, neoadjuvant chemoradiation” OR “treatment, neoadjuvant chemotherapy” OR “treatment, neoadjuvant radiation” OR “treatment, neoadjuvant systemic” OR “neoadjuvant hormone therapy” OR “neoadjuvant targeted therapy” OR Preoperative OR “Preoperative Therap*” OR “Preoperative Treatment*” OR “Primary Systemic Therap*” OR “Primary Therap*” OR “Initial Treatment*” OR “Initial Therap*” OR “Therapeutic Priming” OR “Downstaging Therap*” OR “Tumor Shrinkage Therap*” OR “Novel Auxiliary treatment*” OR “Novel Auxiliary therap*”) |
| 4 | 1 and 2 and 3 |
| **Clinical trial (clinicaltrials.gov/ clinicaltrialsregister.eu)** | |
| No. | Search |
| 1 | (“Breast Neoplasms” OR “breast cancer”) and (“vitamin D” OR “Calcitriol”) and (“Neoadjuvant Therapy” OR “neoadjuvant chemotherapy”) |

**Supplemental Table 2 Data extraction form of included studies**

| **No.** | **Items** |
| --- | --- |
| 1 | Author |
| 2 | Year |
| 3 | Country |
| 4 | Data source |
| 5 | Study design |
| 6 | Observation period, year |
| 7 | Regimen |
| 8 | Age, years |
| 9 | Vitamin D assessment |
| 10 | Vitamin D level, ng/ml |
| 11 | Vitamin D deficiency cut-off value |
| 12 | Vitamin D deficiency, No. (%) |
| 13 | Vitamin D sufficiency, No. (%) |
| 14 | Molecular subtypes |
| 15 | Tumor stage |
| 16 | Outcomes |
| 17 | OR or HR (95%CI) |
| 18 | Sample sizes |

**Supplemental Table 3 Additional baseline characteristics of the six included studies in the meta-analysis**

| **Author** | **Year** | **Observation period, year** | **Regimen** | **Age, years** | **Vitamin D level, ng/ml** | **Vitamin D deficiency,**  **No. (%)** | **Vitamin D sufficiency,**  **No. (%)** |
| --- | --- | --- | --- | --- | --- | --- | --- |
| Clark ^(1)^ | 2014 | NR | Anthracycline,  taxane | 48.1 (±9.0)^a^ | 22.7 (±11.9)^a^ | 34 (41) | 48 (59) |
| Charehbili ^(2)^ | 2016 | 2010-2012 | Neoadjuvant chemotherapy,  ±zoledronic,  vitamin D and calcium | 48.0 (29-68)^b^ | 23.2 (±11.0)^c^ | NR | NR |
| Kim ^(3)^ | 2018 | 2010-2013 | Anthracycline,  cyclophosphamide (AC),  taxane± titanium silicate-1 | 48.7(±9.7)^a^ | 13.0 (3.6-46.3)^b^ | 311 (83) | 63 (17) |
| Viala ^(4)^ | 2018 | 2005-2015 | Anthracycline,  taxane,  trastuzumab | 50.0^d^ | NR | 136 (42) | 191 (58) |
| Atci ^(5)^ | 2021 | 2014-2019 | Doxorubicin,  cyclophosphamide,  paclitaxel,  trastuzumab | 49.7(±12.1) ^a^ | 17.8 (±9.7)^a^ | 38 (43) | 51 (57) |
| Tokunaga ^(6)^ | 2022 | 2009-2019 | Anthracyclines,  taxane,  trastuzumab | 59.0 (28–75)^b^ | 10.7 (3.0-26.6)^b^ | 241 (96) | 9 (4) |

Abbreviations: NR, not reported

^a^, mean (SD); ^b^, median(range); ^c^, median (SD); ^d^, median

unit conversion: vitamin D :1ng/ml=2.5nmol/l

vitamin D deficiency: <20ng/ml; vitamin D sufficiency: ≥20ng/ml

**Supplemental Table 4 Quality assessment of literature on overall pathological response**

| **Authors** | **Year** | **Selection** | | | | **Comparability** | **Outcome** | | | **Score** |
| --- | --- | --- | --- | --- | --- | --- | --- | --- | --- | --- |
|  |  | A | B | C | D | E | F | G | H |  |
| Clark(1) | 2014 | ★ | ★ | ★ | ★ |  | ★ | ★ | ★ | 7 |
| Charehbili(2) | 2016 | ★ | ★ | ★ | ★ |  | ★ | ★ | ★ | 7 |
| Kim(3) | 2018 |  | ★ | ★ | ★ |  | ★ | ★ | ★ | 6 |
| Viala(4) | 2018 | ★ | ★ | ★ | ★ |  | ★ | ★ | ★ | 7 |
| Atci(5) | 2021 |  | ★ | ★ | ★ |  | ★ | ★ | ★ | 6 |
| Tokunaga(6) | 2022 |  | ★ | ★ | ★ |  | ★ | ★ | ★ | 6 |

**Supplemental Table 5 Quality assessment of literature on pathological complete response (pCR)**

| **Authors** | **Year** | **Selection** | | | | **Comparability** | **Outcome** | | | **Score** |
| --- | --- | --- | --- | --- | --- | --- | --- | --- | --- | --- |
|  |  | A | B | C | D | E | F | G | H |  |
| Charehbili(2) | 2016 | ★ | ★ | ★ | ★ |  | ★ | ★ | ★ | 7 |
| Kim(3) | 2018 |  | ★ | ★ | ★ |  | ★ | ★ | ★ | 6 |
| Viala(4) | 2018 | ★ | ★ | ★ | ★ |  | ★ | ★ | ★ | 7 |
| Tokunaga(6) | 2022 |  | ★ | ★ | ★ |  | ★ | ★ | ★ | 6 |

**Supplemental Table 6 Quality assessment of literature on event-free survival (EFS)**

| **Authors** | **Year** | **Selection** | | | | **Comparability** | **Outcome** | | | **Score** |
| --- | --- | --- | --- | --- | --- | --- | --- | --- | --- | --- |
|  |  | A | B | C | D | E | F | G | H |  |
| Clark(1) | 2014 | ★ | ★ | ★ | ★ |  | ★ | ★ | ★ | 7 |
| Kim(3) | 2018 |  | ★ | ★ | ★ | ★★ | ★ | ★ | ★ | 8 |
| Viala(4) | 2018 | ★ | ★ | ★ | ★ | ★★ | ★ | ★ | ★ | 9 |
| Tokunaga(6) | 2022 |  | ★ | ★ | ★ | ★ | ★ | ★ | ★ | 7 |

**Supplemental Method 1 Newcastle-Ottawa Scale risk of bias scoring guide**

**NEWCASTLE - OTTAWA QUALITY ASSESSMENT SCALE COHORT STUDIES**(7)

**Selection**

1) Representativeness of the exposed cohort(8)

a) truly representative of the average _______________ (describe) at multiple sites ★

b) somewhat representative of the average ______________ at multiple sites ★

c) selected group of users e.g. nurses, volunteers

d) no description of the derivation of the cohort

2) Selection of the non-exposed cohort(8)

a) drawn from the same site as the exposed cohort ★

b) drawn from a different source

c) no description of the derivation of the non-exposed cohort

3) Ascertainment of exposure

a) secure record (e.g. surgical records) ★

b) structured interview ★

c) written self-report

d) no description

4) Demonstration that outcome of interest was not present at start of study

a) yes ★

b) no

**Comparability**

1) Comparability of cohorts on the basis of the design or analysis

a) study controls for _____________ (select the most important factor) ★

b) study controls for any additional factor ★(This criteria could be modified to indicate specific control for a second important factor.)

**Outcome**

1) Assessment of outcome

a) independent blind assessment★

b) record linkage★

c) self-report

d) no description

2) Was follow-up long enough for outcomes to occur

a) yes (select an adequate follow up period for outcome of interest) ★

b) no

3) Adequacy of follow up of cohorts

a) complete follow up - all subjects accounted for ★

b) subjects lost to follow up unlikely to introduce bias - small number lost - > ____ % (select an adequate %) follow up, or description provided of those lost) ★

c) follow up rate < ____% (select an adequate %) and no description of those lost

d) no statement

**Supplemental Method 2 The definition of outcomes**

**1. Overall pathological response:** A combination of pathological complete response rate (pCR), residual cancer burden (RCB), and Miller-Payne grading (MPG).


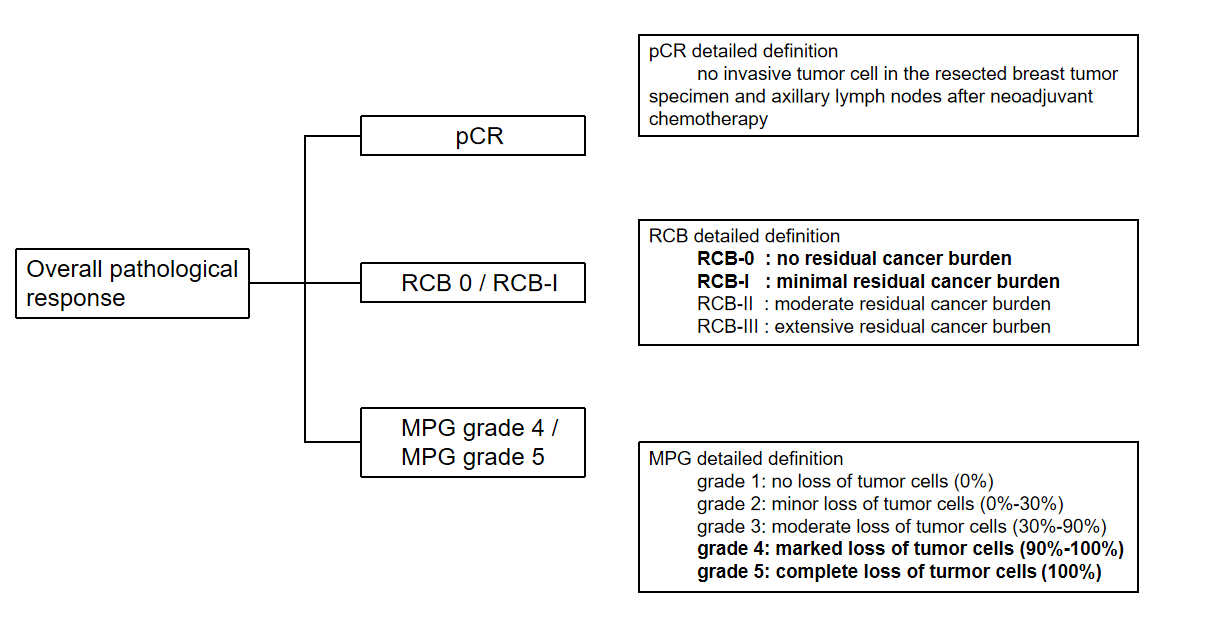


**2. pCR (Pathological complete response)**(2): The absence of invasive tumor cellularity in the resected breast tumor specimen and axillary lymph nodes after neoadjuvant chemotherapy (patients with residual in situ cancer without invasive cancer were considered to have a pCR).

**3.** **RCB (Residual cancer burden)**(9, 10): RCB provides a continuous measurement of the extent of residual cancer, which involves the largest diameter of the invasive cancer, the percent cellularity of the tumor, the number of lymph nodes involved, and the largest diameter of the nodal. A free, interactive, online RCB calculator is available on a web site to calculate the scores (https://www3.mdanderson.org/app/medcalc/index.cfm?pagename=jsconvert3). Cut points are applied to define four RCB classes indicating progressively larger residual disease burden:

RCB-0: RCB score 0, corresponding to pCR defined as no residual invasive carcinoma in the breast and axillary lymph nodes

RCB-I: RCB score 0-1.34; corresponding to minimal residual invasive disease

RCB-II: RCB score 1.34-3.28; corresponding to moderate residual disease

RCB-III: RCB score>3.28; corresponding to extensive residual disease

**4. MPG** **(****Miller/Payne grading)**(11, 12): MPG provides a five-step scale based on tumor cellularity in the excision/mastectomy specimen compared with the pretreatment core biopsy as follows:

grade 1, no reduction in overall cellularity;

grade 2, minor (<30%) loss of cellularity;

grade 3, estimated 30%–90% reduction in tumor cells;

grade 4, >90% loss of tumor cells;

grade 5, no invasive carcinoma (IC); ductal carcinoma in situ may be present.

**5. EFS (Event-free survival)**(13)^:^A combination of progression-free survival (PFS), recurrence-free survival (RFS), disease-free survival (DFS), and time to distant recurrence (TTDR).

**6. PFS (Progression-free survival)**(4)**:** Length of time during and after the treatment of a disease, such as cancer, that a patient lives with the disease but the disease does not get worse.

**7. RFS (Recurrence-free survival)**(14): Period from the end of initial treatment until the disease returns (recurrences) or the patient dies from any cause.

**8. DFS (Disease-free survival)**(3)**:** Period after successful treatment in which there is no appearance of the symptoms or effects of the disease.

**9. TTDR (Time to distant recurrence)**(6): The time from the date of curative surgery to the detection of distant recurrence.

**Supplemental Figure 1A Risk of bias plot on overall pathological response**


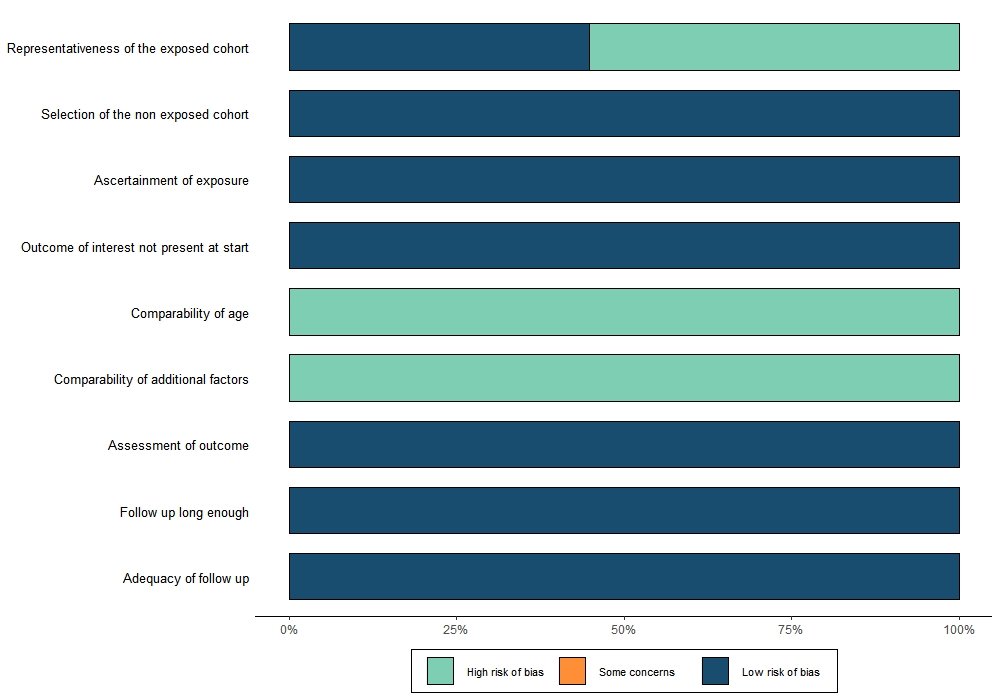


**Supplemental Figure 1B Risk of bias plot on event-free survival (EFS)**


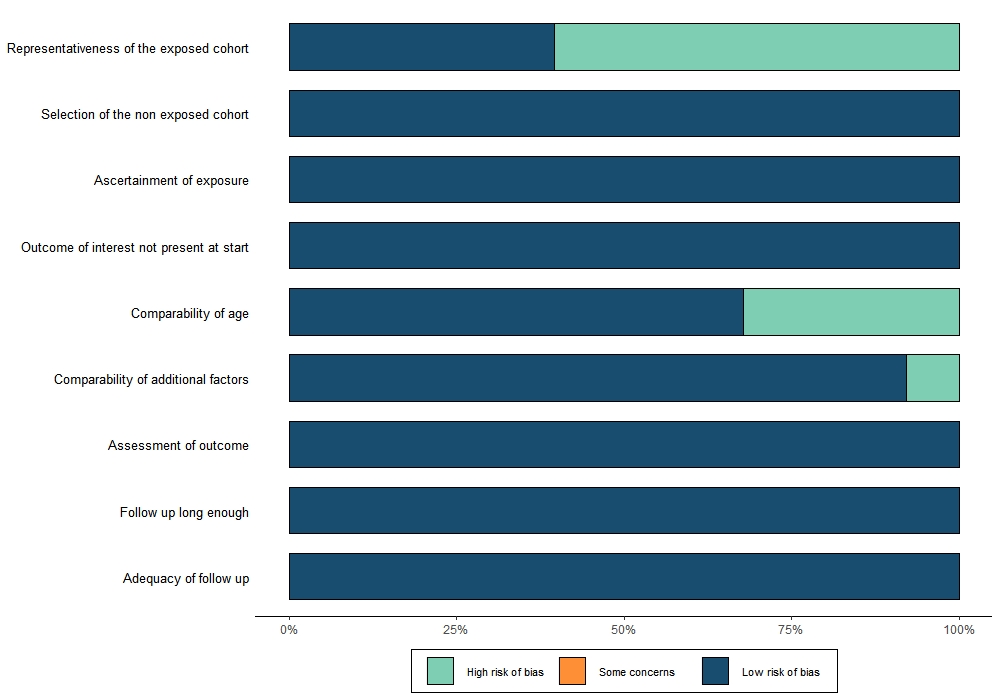


**Supplemental Figure 2A Funnel plot on pathological complete response (pCR)**


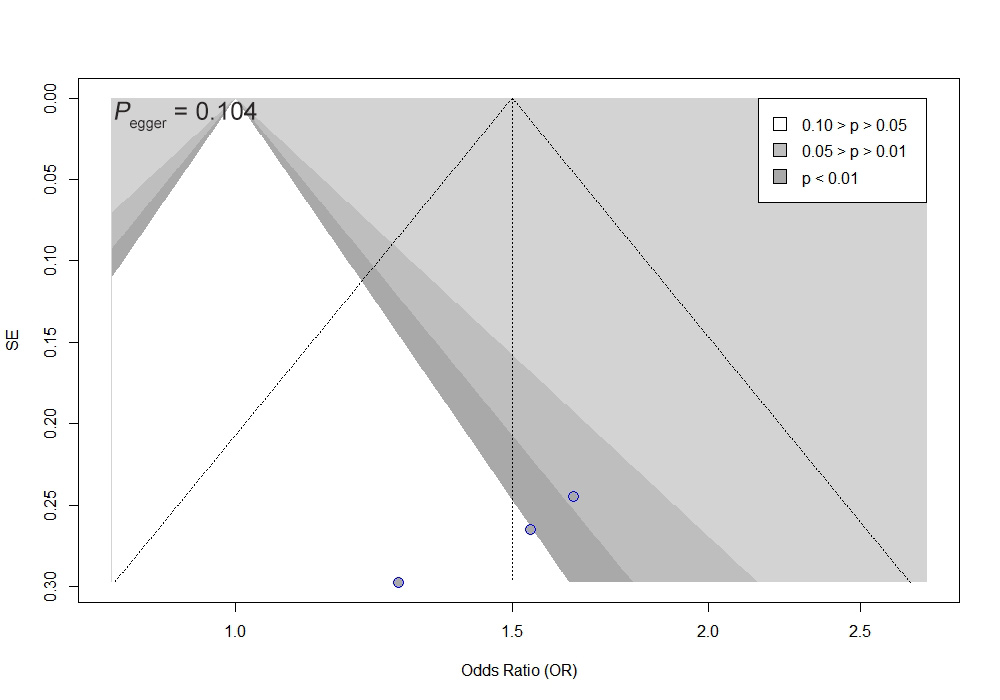


**Supplemental Figure 2B Funnel plot on overall pathological response**


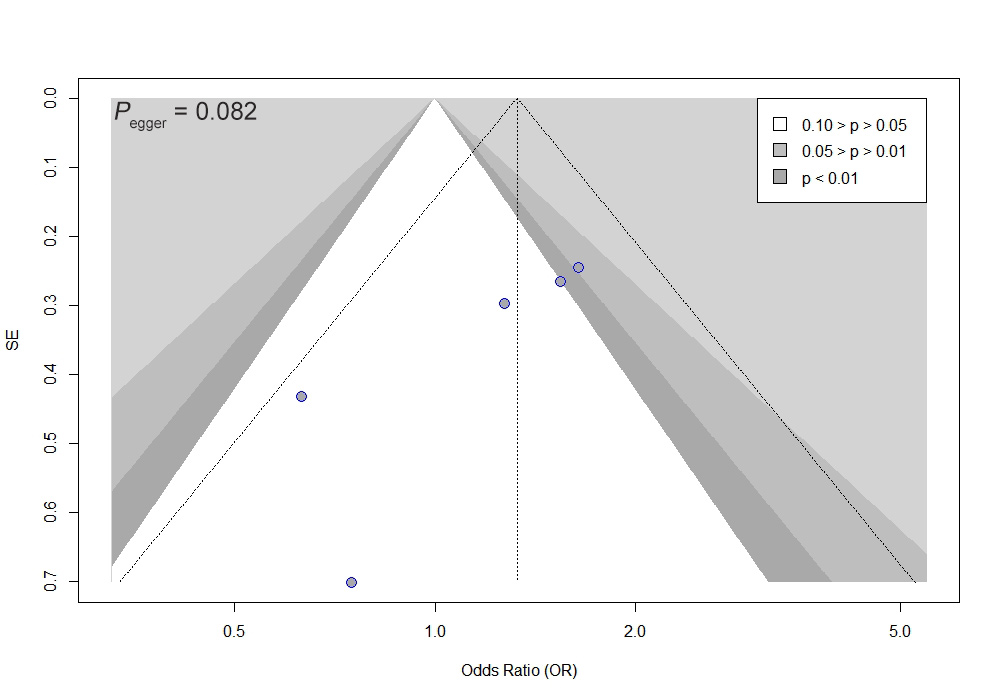


**Supplemental Figure 2C Funnel plot on event-free survival (EFS)**


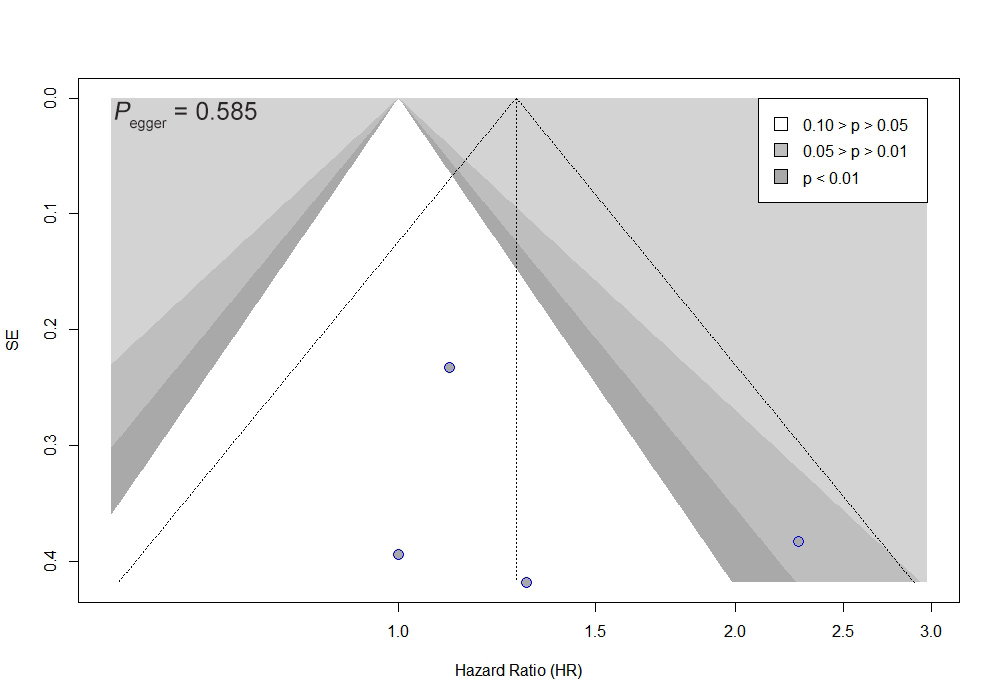


**Supplemental Figure 3 Subgroup analysis on overall pathological response (vitamin D as a continuous variable)**

**
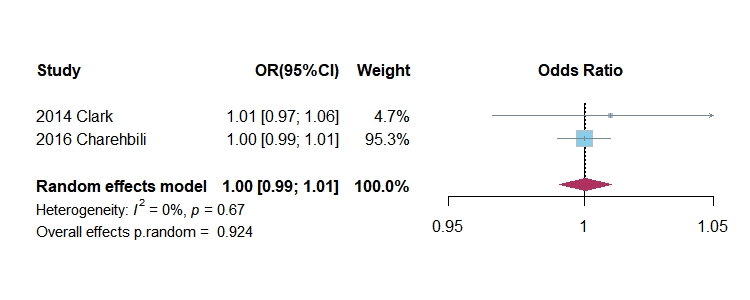
**

**Supplemental Figure 4 Subgroup analysis on event-free survival (EFS)**


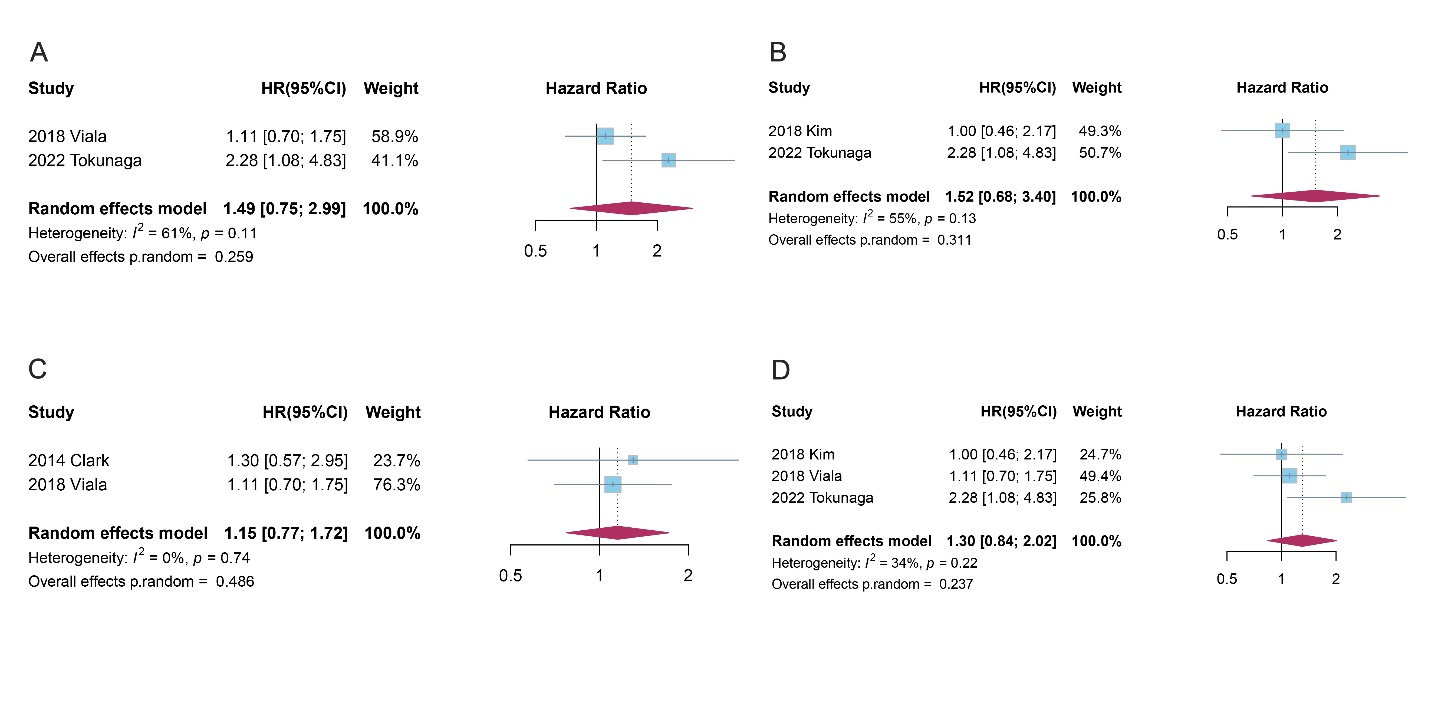


A Subgroup plot with Chemotherapy including trastuzumab, B Subgroup plot with Asian, C Subgroup plot with European, D subgroup plot with Stage I-III

**Supplemental Figure 5A Sensitivity analysis on overall pathological response**


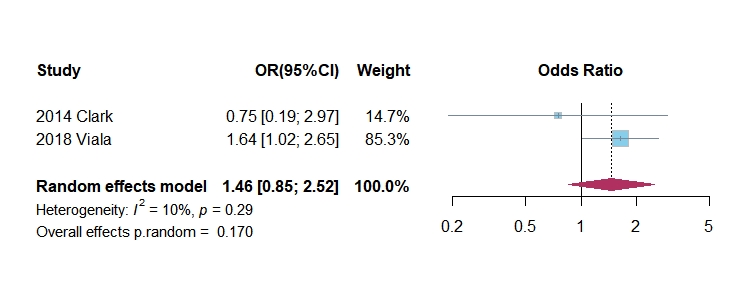


**Supplemental Figure 5B Sensitivity analysis on event-free survival (EFS)**


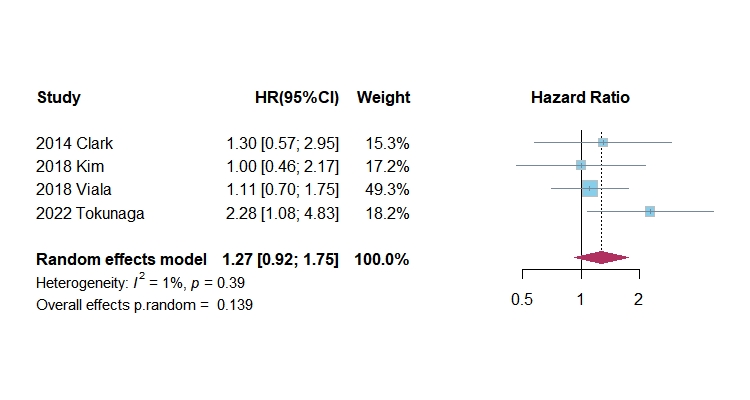


**Supplemental Figure 6 Forest plot of the summarized results regarding overall pathological response (vitamin D as a continuous variable)**

**
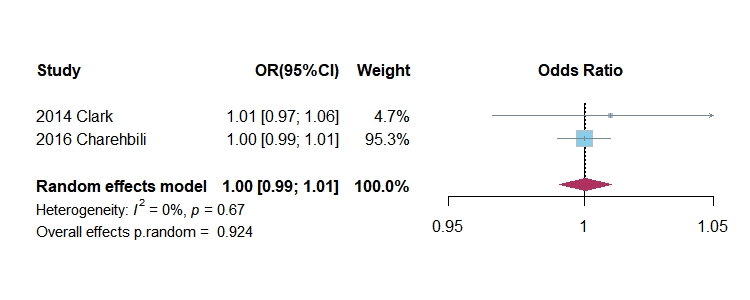
**

**Reference**

1. Clark AS, Chen J, Kapoor S, Friedman C, Mies C, Esserman L, et al. Pretreatment vitamin D level and response to neoadjuvant chemotherapy in women with breast cancer on the I-SPY trial (CALGB 150007/150015/ACRIN6657). Cancer medicine. 2014;3(3):693-701.

2. Charehbili A, Hamdy NAT, Smit VTHBM, Kessels L, van Bochove A, van Laarhoven HW, et al. Vitamin D (25-0H D3) status and pathological response to neoadjuvant chemotherapy in stage II/III breast cancer: Data from the NEOZOTAC trial (BOOG 10-01). Breast (Edinburgh, Scotland). 2016;25:69-74.

3. Kim JS, Haule CC, Kim JH, Lim SM, Yoon KH, Kim JY, et al. Association between Changes in Serum 25-Hydroxyvitamin D Levels and Survival in Patients with Breast Cancer Receiving Neoadjuvant Chemotherapy. Journal of breast cancer. 2018;21(2):134-41.

4. Viala M, Chiba A, Thezenas S, Delmond L, Lamy P-J, Mott SL, et al. Impact of vitamin D on pathological complete response and survival following neoadjuvant chemotherapy for breast cancer: a retrospective study. BMC cancer. 2018;18(1):770.

5. Atci MM, Cekin R. Predictive Importance of Vitamin D on Response to Neoadjuvant Chemotherapy in Turkish Patients with Locally Advanced Hormone Receptor Positive Breast Cancer. Eurasian Journal of Medical Investigation. 2021;5(3):388-93.

6. Tokunaga E, Masuda T, Ijichi H, Tajiri W, Koga C, Koi Y, et al. Impact of serum vitamin D on the response and prognosis in breast cancer patients treated with neoadjuvant chemotherapy. Breast cancer (Tokyo, Japan). 2022;29(1):156-63.

7. Wells GA, Shea B, O'Connell D, Peterson J, Welch V, Losos M, et al. Newcastle-Ottawa Scale for assessing the quality of nonrandomized studies in meta-analyses 2024 [Available from: http://www.ohri.ca/programs/clinical_epidemiology/oxford.asp

8. Stang A. Critical evaluation of the Newcastle-Ottawa scale for the assessment of the quality of nonrandomized studies in meta-analyses. Eur J Epidemiol. 2010;25(9):603-5.

9. Symmans WF, Peintinger F, Hatzis C, Rajan R, Kuerer H, Valero V, et al. Measurement of residual breast cancer burden to predict survival after neoadjuvant chemotherapy. J Clin Oncol. 2007;25(28):4414-22.

10. Peintinger F, Anderson K, Mazouni C, Kuerer HM, Hatzis C, Lin F, et al. Thirty-gene pharmacogenomic test correlates with residual cancer burden after preoperative chemotherapy for breast cancer. Clin Cancer Res. 2007;13(14):4078-82.

11. Ogston KN, Miller ID, Payne S, Hutcheon AW, Sarkar TK, Smith I, et al. A new histological grading system to assess response of breast cancers to primary chemotherapy: prognostic significance and survival. Breast. 2003;12(5):320-7.

12. Wang L, Luo R, Lu Q, Jiang K, Hong R, Lee K, et al. Miller-Payne Grading and 70-Gene Signature Are Associated With Prognosis of Hormone Receptor-Positive, Human Epidermal Growth Factor Receptor 2-Negative Early-Stage Breast Cancer After Neoadjuvant Chemotherapy. Front Oncol. 2021;11:735670.

13. Methods In Medicine CAM. Retracted: Pathologic Complete Response and Its Impact on Breast Cancer Recurrence and Patient's Survival after Neoadjuvant Therapy: A Comprehensive Meta-Analysis. Comput Math Methods Med. 2023;2023:9781535.

14. Hudis CA, Barlow WE, Costantino JP, Gray RJ, Pritchard KI, Chapman JA, et al. Proposal for standardized definitions for efficacy end points in adjuvant breast cancer trials: the STEEP system. J Clin Oncol. 2007;25(15):2127-32.
