# Supplementary material for: Pretreatment plasma vitamin D and response to neoadjuvant chemotherapy in breast cancer: evidence from pooled analysis of cohort studies
Source: Int J Surg. 2024 Nov 18;110(12):8126–35. doi: 10.1097/JS9.0000000000002142 (PMC11634150; doi:10.1097/JS9.0000000000002142)
Supplement: SUPPLEMENTARY MATERIAL [file js9-110-8126-s003.docx]

**Identification of studies via databases and registers**

**Identification**

Records removed before screening:

Duplicate records removed

(n=46)

Records identified from: Databases

Medline (n=17)

Embase (n=107)

Cochrane Library(n=10)

Web of Science (n=43)

Google Scholar (n=2)

Registers

Clinical Trial (n=10)

**Screening**

Records excluded

Irrelevant (n=112)

Conference abstract (n=19)

Commentary (n=1)

Records screened

(n=143)

Reports sought for retrieval

(n=11)

Reports not retrieved

(n=0)

Reports excluded:

Inadequate outcome data

(n=3)

Overlapped population

(n=1)

Insufficient sample size

(n=1)

Reports assessed for eligibility (n=11)

Studies included in meta-analysis (n=6)

**Included**

*From:* Page MJ, McKenzie JE, Bossuyt PM, Boutron I, Hoffmann TC, Mulrow CD, et al. The PRISMA 2020 statement: an updated guideline for reporting systematic reviews. BMJ 2021;372:n71. doi: 10.1136/bmj.n71
